# Revision of a nonunited subtrochanteric femoral fracture around a failed intramedullary nail with the use of RIA products, BMP-7 and hydroxyapatite: a case report

**DOI:** 10.1186/1752-1947-5-87

**Published:** 2011-03-01

**Authors:** Christopher Tzioupis, Pavlos Panteliadis, Zakareya Gamie, Eleftherios Tsiridis

**Affiliations:** 1 Academic Department of Trauma and Orthopaedics, School of Medicine, University of Leeds, Leeds General Infirmary, Leeds Teaching Hospitals NHS Trust, Clarendon Wing A, Great George Street, Leeds, LS1 3EX, UK; 2 Academic Orthopaedic Unit, Faculty of Medicine, Aristotle University of Thessaloniki 541 24, Greece

## Abstract

**Introduction:**

Femoral subtrochanteric fractures are commonly treated using intramedullary devices. Failure of the implant and subsequent nonunion is still an issue, however, and limited evidence exists regarding the most appropriate treatment.

**Case presentation:**

We report the case of an 80-year-old Caucasian woman with a subtrochanteric fracture originally treated using a trochanteric gamma nail which failed, resulting in a nonunion and fracture of its proximal end. The nonunion was revised with the removal of the broken trochanteric gamma nail, application of a condylar blade plate, ipsilateral Reamer/Irrigator/Aspirator autografting, recombinant human bone morphogenetic protein-7 and injectable hydroxyapatite cement. The fracture united fully at ten months following revision surgery, with no signs of femoral head avascular necrosis at 18-month follow-up.

**Conclusion:**

The essential requirements for success when revising a nonunited fracture are to provide anatomical reduction, mechanical stability, bone defect augmentation and biological stimulation to achieve healing. Current advances in molecular biology, such as recombinant human bone morphogenetic protein-7, and biotechnology such as the Reamer/Irrigator/Aspirator system and hydroxyapatite injectable cement can improve patient outcomes over the use of our traditional revision techniques.

## Introduction

Most fractures of the subtrochanteric region of the femur heal when treated using contemporary methods of internal fixation [1]. Improved understanding of the biomechanics of this region has shifted treatment toward the use of intramedullary devices (IMD) as the shorter-levered arm on the proximal fixation results in greater load sharing and less bending movement across the fracture and implant [2,3], reducing the rate of implant failure [2,4]. The overall incidence of failure for any type of fixation and subsequent nonunion of subtrochanteric fractures varies from 7% to 20% [5]. Complications occur mainly in patients with poor bone quality, unfavorable fracture patterns and suboptimal positioning of the fixation implant [1,5]. IMD complications include femoral shaft fracture below the tip of the IMD, collapse of the fracture and cutting out of the femoral neck screw, for which reoperation is required [6]. For extramedullary devices such as the sliding hip screw or the dynamic condylar screw, failure often occurs following screw cutout [2,3].

There is limited evidence regarding the most appropriate method of treating a nonunion of a subtrochanteric fracture [1,3]. Debridement of fibrous tissue, correction of varus malalignment, autografting and fracture compression are essential to achieve union [5]. It has been reported that subtrochanteric nonunions treated with the condylar blade plate (CBP) are associated with good healing rates [1,5]. Autograft harvesting from the iliac crest, however, is related to comorbidities [7], increasing the need for autograft substitution. The Reamer/Irrigator/Aspirator (RIA) system (Synthes North America, Inc., West Chester, PA, USA) is a recently developed device used to perform corticocancellous intramedullary autografts containing human mesenchymal stem cells (hMSCs) to stimulate bone healing [8]. In addition, recombinant human bone morphogenetic protein-7 (rhBMP-7) has been introduced with success for the treatment of nonunions [9]. Biocompatible materials such as hydroxyapatite (HA) have also been tested in combination with rhBMP-7 *in vivo *to induce osteogenic differentiation of hMSCs [10]. We report the case of a patient with a subtrochanteric fracture originally treated using a trochanteric gamma nail (TGN) (Gamma 3 IM nailing system; Stryker Biotech, Hopkinton, MA, USA) which failed and resulted in a nonunion and fracture of the proximal end of the TGN device. The nonunion was revised with the removal of the broken TGN, application of a CBP, ipsilateral RIA autografting, and use of BMP-7 and HA injectable cement, with success and healing achieved at 10 months following revision surgery.

## Case presentation

**Figure 1:**
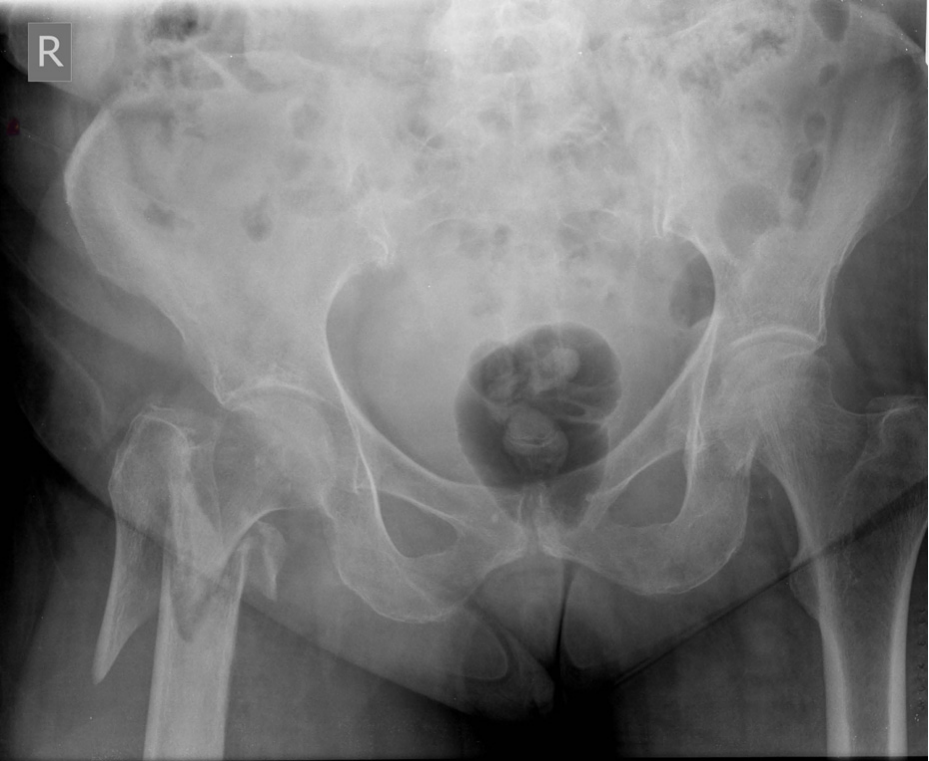
**Anteroposterior radiograph of the pelvis demonstrating a right subtrochanteric femoral fracture classified as 31-A3.3 under the AO Foundation (AO)/Orthopaedic Trauma Association (OTA) fracture classification system**.

**Figure 2:**
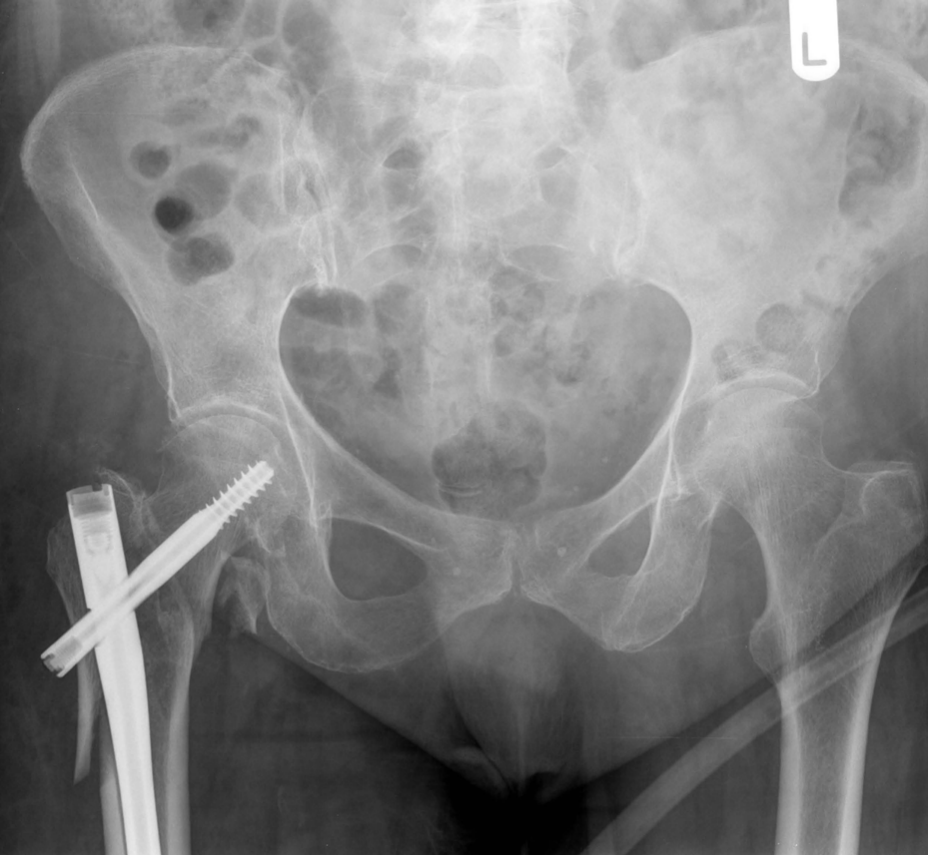
**Anteroposterior radiograph demonstrating reduction and stabilization of the fracture with a trochanteric gamma nail (TGN)**.

**Figure 3:**
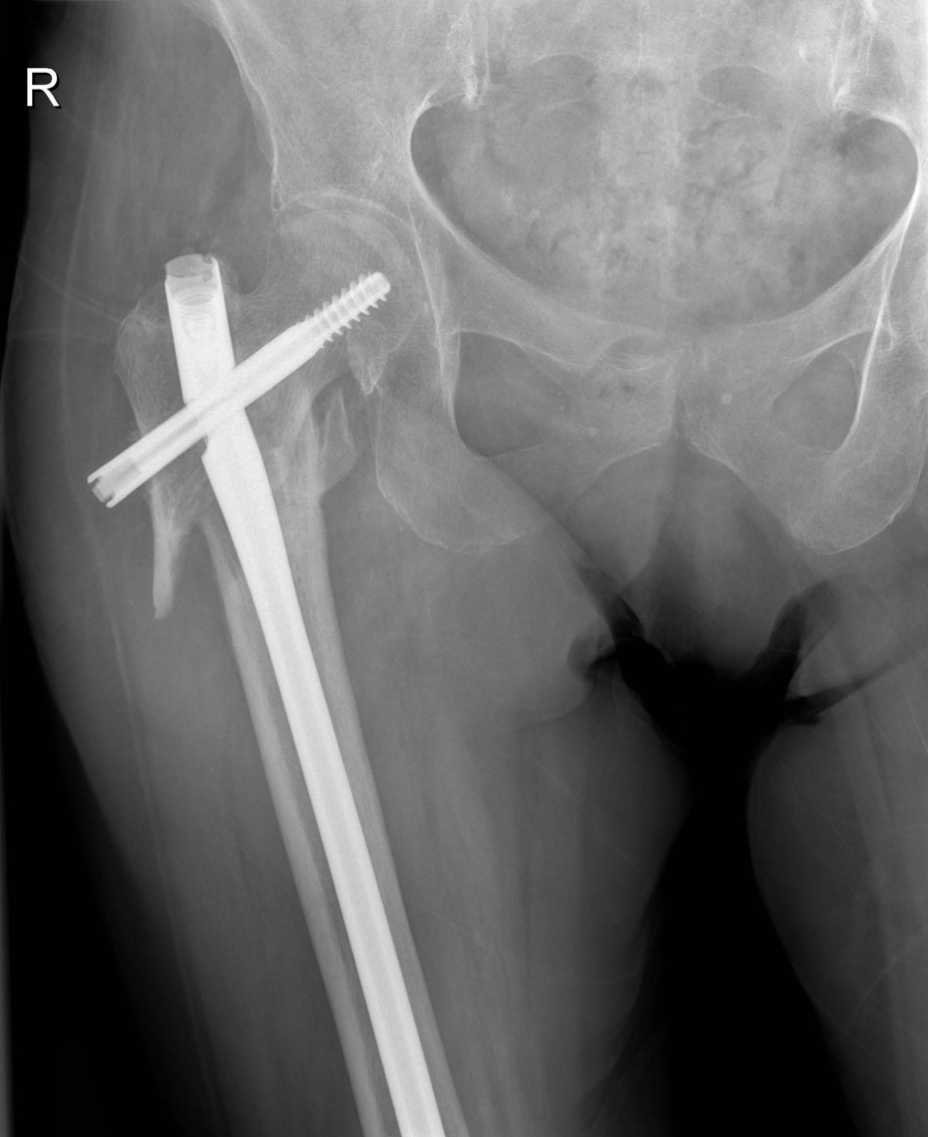
**Anteroposterior radiograph demonstrating failure of the TGN with fracture of the proximal end of the nail, nonunion of the fracture site and varus deformity of the proximal femur**.

**Figure 4:**
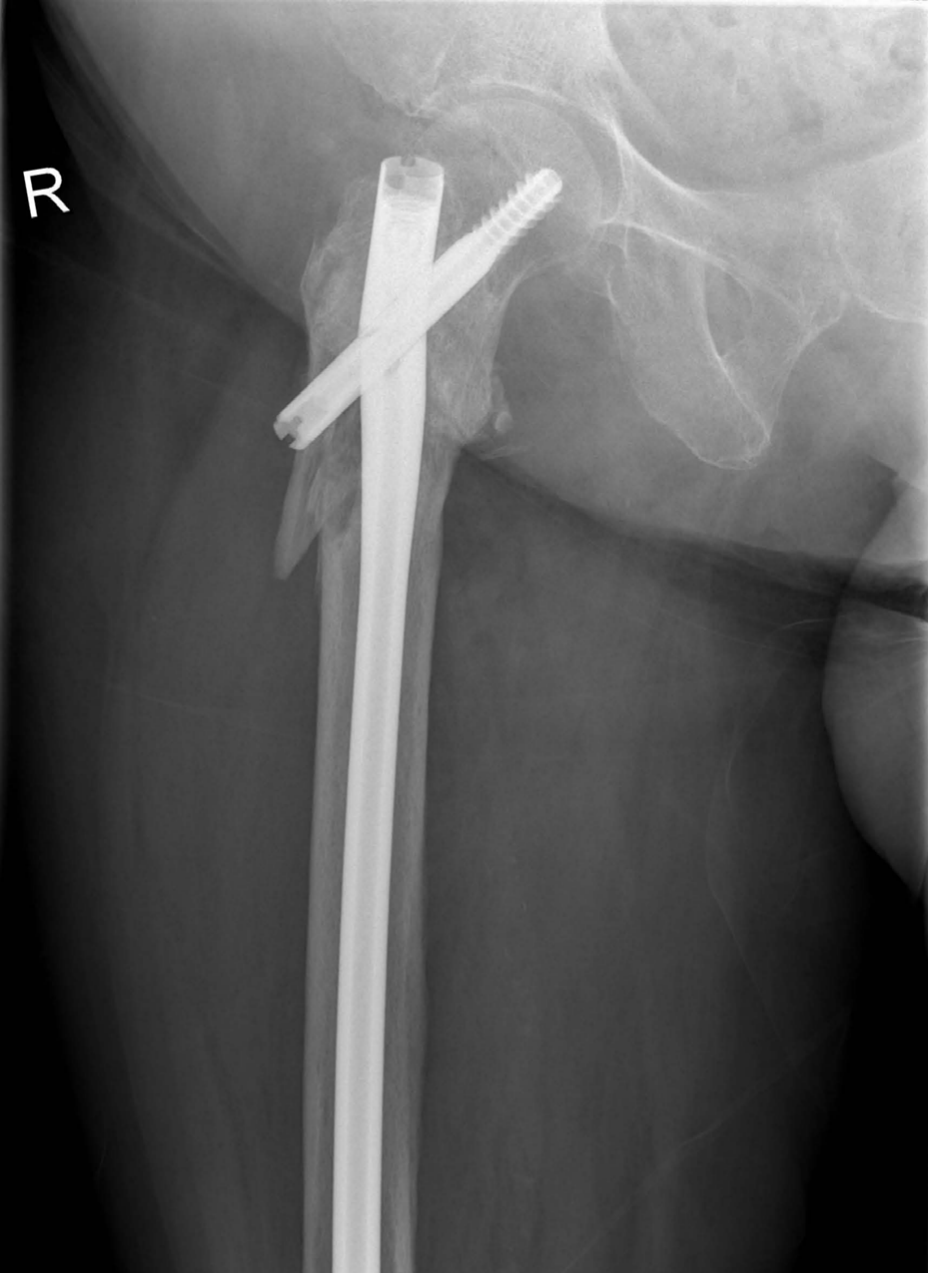
**Lateral radiograph demonstrating failure of the TGN**.

**Figure 5:**
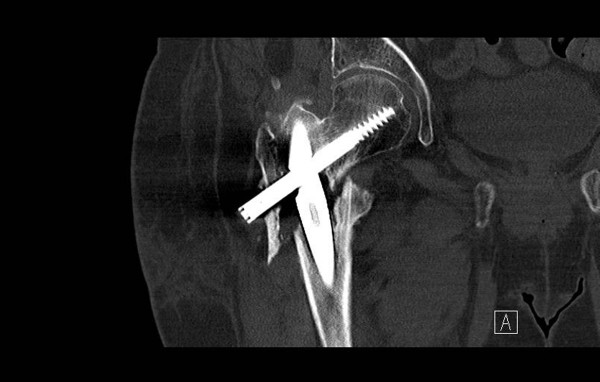
**Coronal computed tomographic scan confirming the diagnosis of nonunion at the fracture site**.

An 80-year-old Caucasian woman sustained a right subtrochanteric femoral fracture following a domestic fall, classified according to the AO Foundation (AO)/Orthopaedic Trauma Association (OTA) fracture classification system as 31-A3.3 (Figure 1). The fracture was reduced and stabilized with a TGN (Figure 2). The patient had an uncomplicated recovery and was discharged to home. After three months, the patient reported pain on ambulation, and radiographs failed to demonstrate sufficient callus formation. Subsequent radiographs obtained at four and six months revealed delayed union; therefore, the nail was dynamized by removing the two distal locking screws to promote union. At 10 months following revision surgery, the patient's pain had increased, making her unable to bear weight, and at that time a further radiograph revealed failure of the TGN with fracture of the proximal end of the nail, nonunion of the fracture site and varus deformity of the proximal femur (Figures 3 and 4). A computed tomographic scan confirmed the diagnosis of nonunion (Figure 5), and revision surgery was planned to remove the failed TGN and to stabilize the fracture with an extramedullary device and graft.

**Figure 6:**
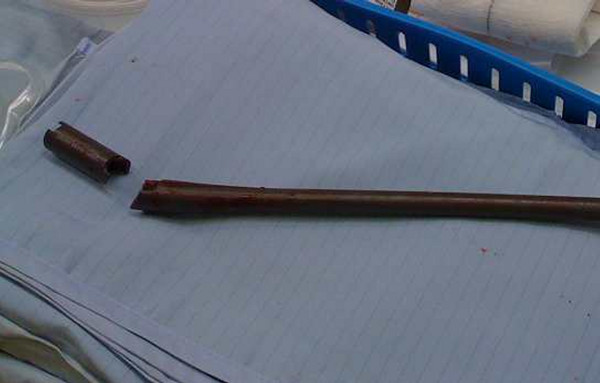
**TGN with fracture of the proximal end of the nail**.

**Figure 7:**
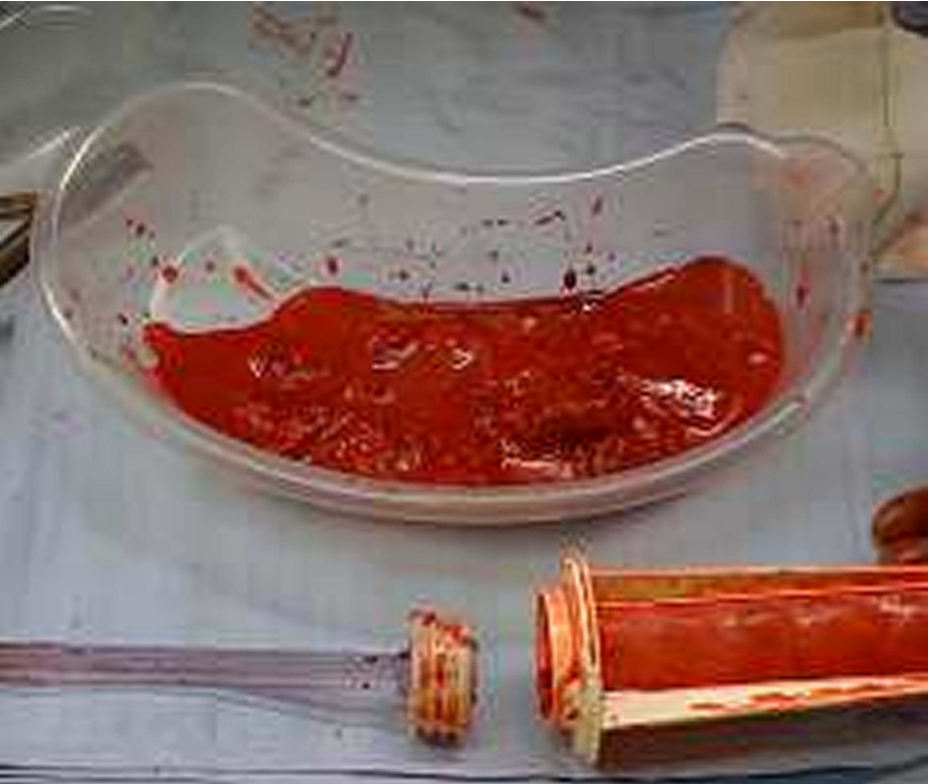
**Reamer/Irrigator/Aspirator aspirate**.

**Figure 8:**
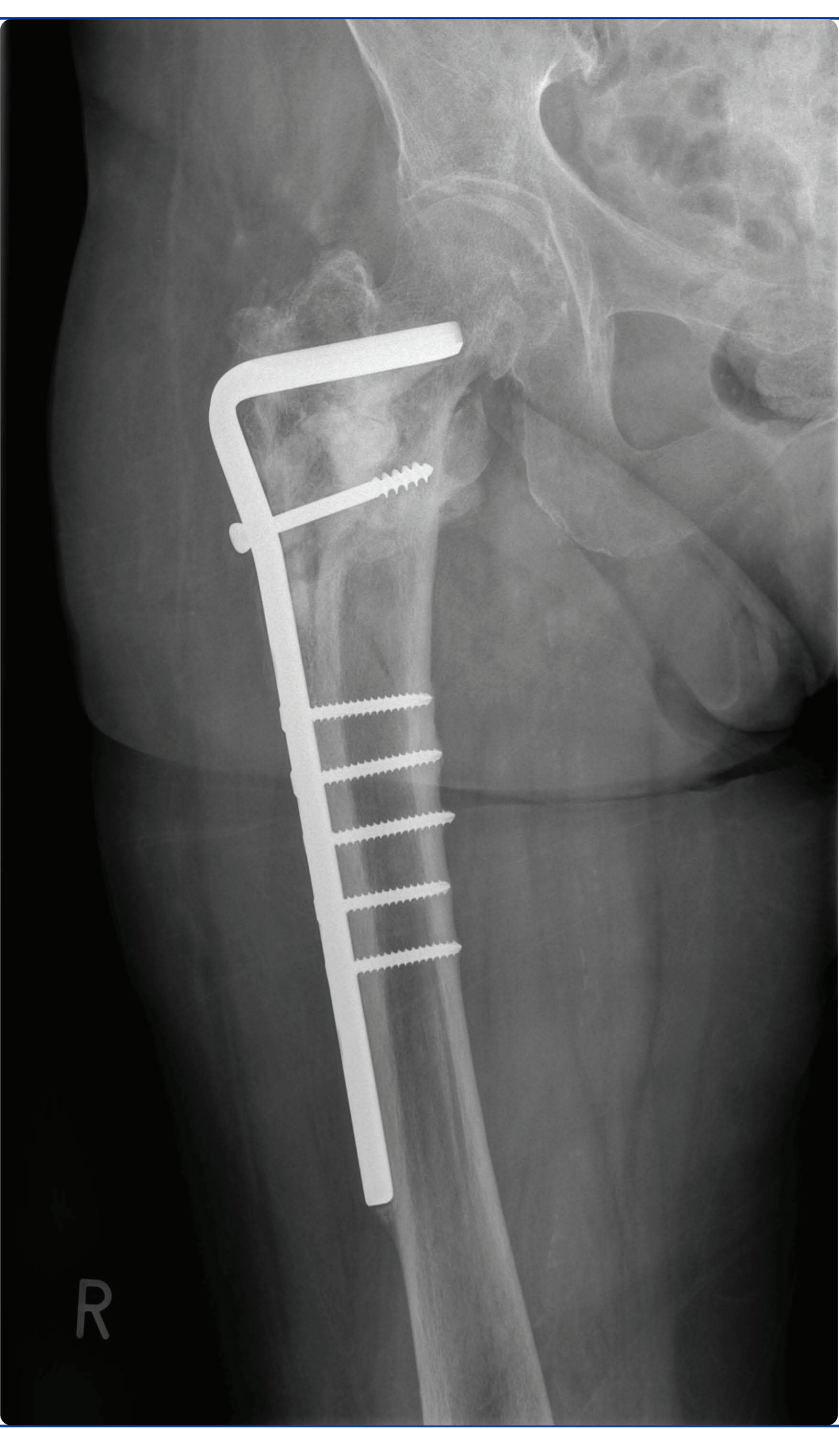
**Anteroposterior radiograph demonstrating the 90° condylar blade plate (CBP) restoring the proper shaft-neck hip angle and union of the fracture site at 10 months following revision surgery with no signs of avascular necrosis of the femoral head**.

**Figure 9:**
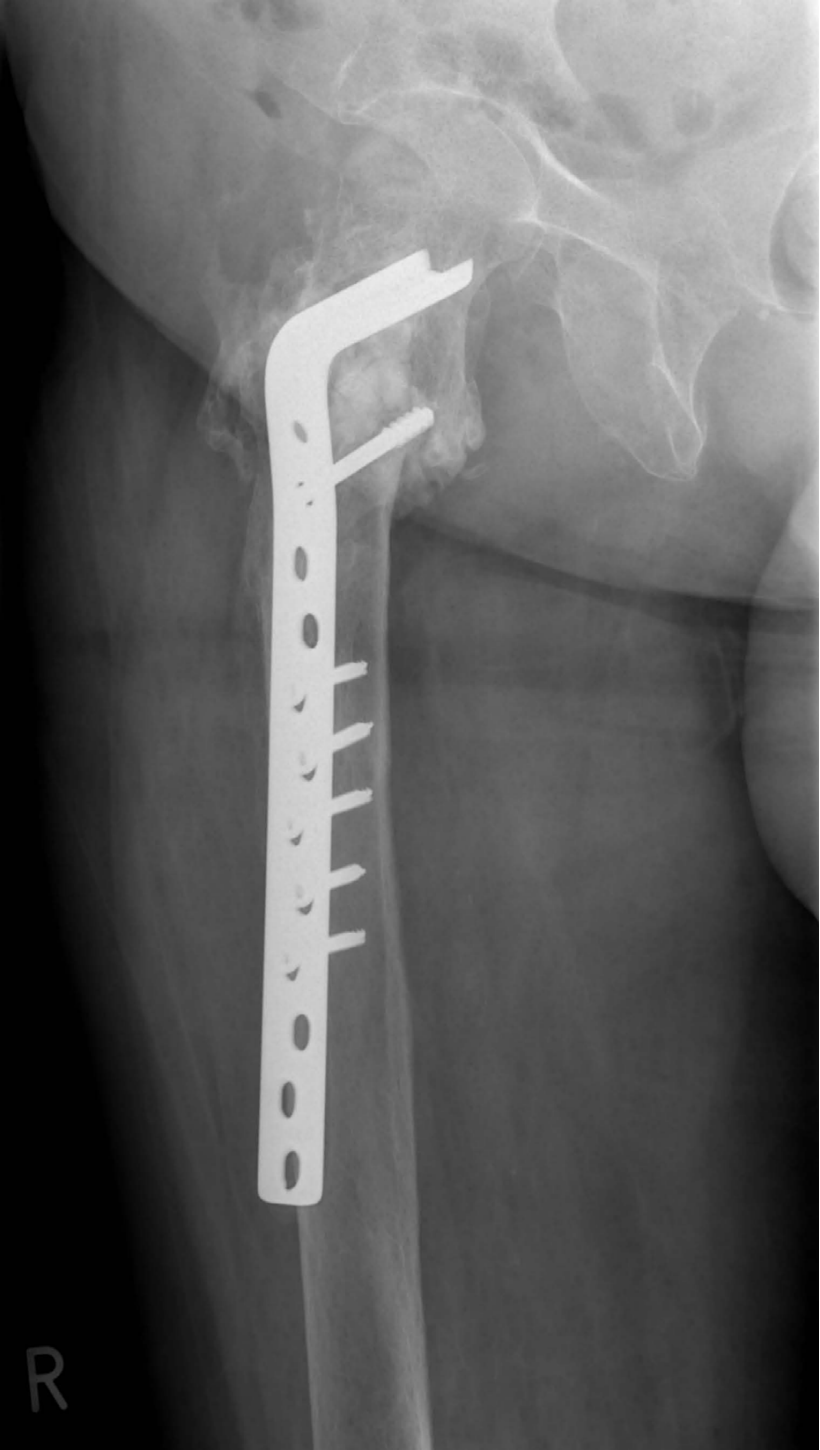
**Lateral radiograph demonstrating the 90° CBP restoring the proper shaft-neck hip angle and union of the fracture site at 10 months following revision surgery with no signs of avascular necrosis of the femoral head**.

The patient was placed in a lateral decubitus position without traction on a radiolucent table. Four hundred milligrams of teicoplanin were administered preoperatively according to the standard antibiotic prophylaxis protocol for revision trauma surgery at our institution. The old incision was incorporated and extended distally into a straight lateral approach to the femur with the fracture site fully exposed. The broken TGN was removed through the fracture site, and the fibrous nonunion tissue was taken out until bleeding bone was exposed (Figure 6). Care was taken to protect the vascular supply to the fracture site by minimal muscle stripping. Six tissue samples were sent for microbiological testing to exclude infection according to revision surgery protocol. The fracture was then aligned over an intramedullary guidewire for reaming. The RIA reamers were used to ream and irrigate the endosteal bone-implant interface, and thereafter intramedullary corticocancellous reaming autograft was collected following the standard RIA protocol (Figure 7). Reduction forceps were then used to accurately reduce the fracture in the desired anatomical position, and guidewires were placed to determine the direction and starting point for the CBP insertion. A 90° CBP was inserted, restoring the proper shaft-neck hip angle compared to the contralateral site (Figures 8 and 9). Prior to CBP insertion, the femoral neck was filled with injectable HA cement (BoneSource BVF; Stryker Biotech) to fill the void created by the removal of the proximal TGN screw and augment its mechanical strength. The RIA autograft was mixed with the rhBMP-7 implant (Stryker Biotech) and added onto the fracture site.

Postoperatively, the patient was administered low-molecular-weight heparin prophylaxis for six weeks. Partial weight bearing was commenced from the second postoperative week onward as dictated by the patient's tolerance of pain. Clinical and radiographic follow-up was arranged at 6 weeks and 3, 6, 12 and 18 months. The fracture united fully at 10 months following revision surgery, with no sign of femoral head avascular necrosis at the 18-month follow-up examination. The patient achieved a full range of hip movement, scoring 80 on the Charnely D'Aubigne Postel scale [11].

## Discussion

There has been controversy in the literature regarding the best type of implant for the fixation of subtrochanteric femoral fractures [2]. Both intramedullary and extramedullary devices have been advocated for the management of subtrochanteric fractures [3]. Less favorable results and implant failure occur in patients with osteoporotic bone, complex fracture patterns, suboptimal implant positioning, shaft medialization and varus malreduction, for which revision fixation may be recommended [1,2,5,12]. The biomechanical advantages of IMD are often diminished by suboptimal fracture reduction and false entry point prior to nail insertion [5]. The incidence of neck screw cutout and fracture below the nail was found to be 4% and 3.2%, respectively, for the TGN nail in a comparison study with the proximal femoral nail (PFN) [13]. The PFN was associated with varus malreduction in 7.2% of patients and screw migration resulting in fracture collapse in 8% of patients; however, with a lower incidence of shaft fractures and neck screw cut-out incidence, compared to TGN [13]. In a prospective study comparing the success rate of TGNs, PFNs and dynamic hip screws for unstable trochanteric fractures, the TGN group had four failures in 40 patients attributed to screw cutout and nonunion, which was greater than the number of failures in the other groups studied [6].

In a recent systematic review, pooled analysis of level I studies suggested a nonsignificant lower risk of failure in the IMD group compared with extramedullary devices and no difference in the rate of nonunion [2]. Modes of failure included femoral fracture in the IMD group and screw cutout in the extramedullary device group. Another frequent mode of failure in the dynamic condylar screw (DCS) implant group was fracture of the plate through the proximal screw hole due to inadequate restoration of the medial calcar and fatigue loading of the DCS implant [2]. It is therefore important to restore the medial column to prevent cyclical loading of the plate on the tension side of the femur and potentially implant failure. This study also highlighted a lack of agreement regarding the definition of a subtrochanteric fracture. It has been defined as a fracture occurring at the level of the lesser trochanter or approximately 5 cm below it [2]. Classification systems have also included intertrochanteric fractures with distal extension into the subtrochanteric region, such as reverse obliquity intertrochanteric fractures [2]. However, the AO/OTA classification system has classified these types of fractures separately under 31-A3, and they have been included in other studies and the current case report because of the rare occurrence of a pure subtrochanteric fracture.

The revision of a nonunited subtrochanteric fracture is challenging because of the varus deformity of the proximal fragment, bone loss and comminution, and occasionally by the failed previous implant [1,5]. Currently, there is no strong evidence to support the use of either IMD or extramedullary devices in the revision of a failed subtrochanteric nonunion [1]. However, the CBP has been advocated for fractures with a very short proximal fragment and large deformities or defects in the region of the piriformis fossa and greater trochanter entry site [1]. The CBP is able to target the area below the femoral head that is unlikely to be compromised by the previous fixation.

In our present case, we elected to revise the failed TGN device with an extramedullary CBP to provide anatomical reduction and fracture site compression, as bone loss and the proximity of the fracture to the femoral neck would not have allowed the insertion of a revision nail to achieve these successfully [1,5]. Previous reports in the literature have confirmed the limited capacity of an IMD to correct the alignment and compress subtrochanteric nonunions to healing in a surgical revision, which are advantages that a CBP can offer [5,14]. This added advantage was protected by augmenting the bone biology. The combination of RIA autograft, BMP-7 and HA cement was used because of the patient's bone loss and to restore the medial column to prevent cyclical loading of the plate on the tension side of the femur and potentially implant failure.

The gold standard for enhancing bone healing in nonunited fractures is an autologous bone graft [7]; however, this procedure has been associated with donor site morbidity and limited availability [7]. The RIA system was developed originally as a simultaneous reaming and aspiration system to reduce intramedullary pressure, heat generation and possibly fat embolism [15]. In addition, it has been recently reported that RIA aspirate contains hMSCs [16], which are known to differentiate toward the osteogenic lineage under the appropriate stimuli [10,17].

Removing the TGN and proximal screw, as well as the fibrous tissue, from the nonunion site left a significant bone defect to be filled in our surgical revision case. Using intramedullary RIA reamings and BMP-7 was considered appropriate, as RIA reamings were available through the fracture site, avoiding the potential hazards of iliac crest harvesting. Furthermore, BMP-7 has previously been used with success in randomized human nonunion studies [9] and in experimental healing of metaphyseal bone defects [18]. In addition, the injected HA cement provided temporary mechanical support to the subchondral zone of the femoral head after removal of the proximal TGN screw, as the CBP blade did not reach this zone [19].

## Conclusion

To the best of our knowledge, this is the first case study to report the successful combination of RIA autograft, BMP-7 and HA cement for the treatment of an established subtrochanteric nonunion. The essential requirements for success when revising a nonunited fracture are to provide anatomical reduction, mechanical stability, bone defect augmentation and biological stimulation to achieve healing. Current advances in molecular biology, such as rhBMP-7, and biotechnology, such as the RIA system and HA injectable cement, can improve the outcomes of patients over the use of our traditional surgical revision techniques.

## Abbreviations

CBP: condylar blade plate; HA: hydroxyapatite; hMSCs: human mesenchymal stem cells; IMD: intramedullary device; rhBMP-7: recombinant human bone morphogenetic protein-7; RIA: Reamer/Irrigator/Aspirator; TGN: trochanteric gamma nail.

## Consent

Written informed consent was obtained from the patient for publication of this case report and accompanying images. A copy of the written consent is available for review by the Editor-in-Chief of this journal.

## Competing interests

The authors declare that they have no competing interests.

## Authors' contributions

CT reviewed the literature and was involved in manuscript preparation and editing. PP reviewed the literature, wrote a first draft of the manuscript and was involved in manuscript preparation and editing. ZG reviewed the literature and was involved in manuscript preparation, editing and submission. ET carried out the surgical procedure and was involved with the conception of the report, reviewed the literature, corrected and finalised the manuscript. All authors read and approved the final manuscript.

## References

[B1] Haidukewych GJ Nonunion of fractures of the subtrochanteric region of the femur Tech Orthop 2008 23 131 136 10.1097/BTO.0b013e31817bf280 15021152

[B2] Kuzyk PR Bhandari M McKee MD Russell TA Schemitsch EH Intramedullary versus extramedullary fixation for subtrochanteric femur fractures J Orthop Trauma 2009 23 465 470 10.1097/BOT.0b013e3181acfdfd 19550236

[B3] Parker MJ Handoll HH Gamma and other cephalocondylic intramedullary nails versus extramedullary implants for extracapsular hip fractures in adults Cochrane Database Syst Rev 2008 3 CD000093 18646058 10.1002/14651858.CD000093.pub4

[B4] Shukla S Johnston P Ahmad MA Wynn-Jones H Patel AD Walton NP Outcome of traumatic subtrochanteric femoral fractures fixed using cephalo-medullary nails Injury 2007 38 1286 1293 10.1016/j.injury.2007.05.013 17981282

[B5] De Vries JS Kloen P Borens O Marti RK Helfet DL Treatment of subtrochanteric nonunions Injury 2006 37 203 211 10.1016/j.injury.2005.09.017 16417905

[B6] Papasimos S Koutsojannis CM Panagopoulos A Megas P Lambiris E A randomised comparison of AMBI, TGN and PFN for treatment of unstable trochanteric fractures Arch Orthop Trauma Surg 2005 125 462 468 10.1007/s00402-005-0021-5 16059696

[B7] Arrington ED Smith WJ Chambers HG Bucknell AL Davino NA Complications of iliac crest bone graft harvesting Clin Orthop Relat Res 1996 329 300 309 10.1097/00003086-199608000-00037 8769465

[B8] Porter RM Liu F Pilapil C Betz OB Vrahas MS Harris MB Evans CH Osteogenic potential of reamer irrigator aspirator (RIA) aspirate collected from patients undergoing hip arthroplasty J Orthop Res 2009 27 42 49 10.1002/jor.20715 18655129 PMC2648608

[B9] Friedlaender GE Perry CR Cole JD Cook SD Cierny G Muschler GF Zych GA Calhoun JH LaForte AJ Yin S Osteogenic protein-1 (bone morphogenetic protein-7) in the treatment of tibial nonunions J Bone Joint Surg Am 2001 83-A Suppl 1 S151 S158 11314793 PMC1425155

[B10] Tsiridis E Ali Z Bhalla A Heliotis M Gurav N Deb S DiSilvio L In vitro and in vivo optimization of impaction allografting by demineralization and addition of rh-OP-1 J Orthop Res 2007 25 1425 1437 10.1002/jor.20387 17557338

[B11] Charnley J The long-term results of low-friction arthroplasty of the hip performed as a primary intervention J Bone Joint Surg Br 1972 54 61 76 5011747

[B12] Haidukewych GJ Israel TA Berry DJ Reverse obliquity fractures of the intertrochanteric region of the femur J Bone Joint Surg Am 2001 83-A 643 650 11379732 10.2106/00004623-200105000-00001

[B13] Herrera A Domingo LJ Calvo A Martínez A Cuenca J A comparative study of trochanteric fractures treated with the Gamma nail or the proximal femoral nail Int Orthop 2002 26 365 369 10.1007/s00264-002-0389-6 12466870 PMC3620972

[B14] Rahme DM Harris IA Intramedullary nailing versus fixed angle blade plating for subtrochanteric femoral fractures: a prospective randomised controlled trial J Orthop Surg (Hong Kong) 2007 15 278 281 18162669 10.1177/230949900701500306

[B15] Giannoudis PV Tzioupis C Green J Surgical techniques: how I do it? The Reamer/Irrigator/Aspirator (RIA) system Injury 2009 40 1231 1236 10.1016/j.injury.2009.07.070 19783249

[B16] Porter RM Ivkovic A Wells J Glatt V Harris MB Vrahas MS Evans C Characterization and utilization of mesenchymal progenitor cells recovered with the Reamer-Irrigator-Aspirator [Abstract] Eur Cell Mater 2008 16 Suppl 2 19

[B17] Belthur MV Conway JD Jindal G Ranade A Herzenberg JE Bone graft harvest using a new intramedullary system Clin Orthop Relat Res 2008 466 2973 2980 10.1007/s11999-008-0538-3 18841433 PMC2628246

[B18] Tsiridis E Morgan EF Bancroft JM Song M Kain M Gerstenfeld L Einhorn TA Bouxsein ML Tornetta P Effects of OP-1 and PTH in a new experimental model for the study of metaphyseal bone healing J Orthop Res 2007 25 1193 1203 10.1002/jor.20420 17506507

[B19] Tsiridis E Ali Z Bhalla A Gamie Z Heliotis M Gurav N Deb S DiSilvio L In vitro proliferation and differentiation of human mesenchymal stem cells on hydroxyapatite versus human demineralised bone matrix with and without osteogenic protein-1 Expert Opin Biol Ther 2009 9 9 19 10.1517/14712590802622473 19063689

